# Conformational Changes of Acyl Carrier Protein Switch the Chain Length Preference of Acyl-ACP Thioesterase ChFatB2

**DOI:** 10.3390/ijms24076864

**Published:** 2023-04-06

**Authors:** Tianxiang Yang, Yunlong Yang, Ming Yang, Jiangang Ren, Changying Xue, Yanbin Feng, Song Xue

**Affiliations:** School of Bioengineering, Dalian University of Technology, Dalian 116023, China

**Keywords:** acyl carrier protein, thioesterase, selectivity, conformational change, circular dichroism

## Abstract

Microbial fatty acids are synthesized by Type II fatty acid synthase and could be tailored by acyl-ACP thioesterase. With the prospects of medium-chain fatty-acid-derivative biofuels, the selectivity of thioesterase has been studied to control the fatty acid product chain length. Here, we report an alternative approach by manipulating the acyl carrier protein portion of acyl-ACP to switch the chain length propensity of the thioesterase. It was demonstrated that ChFatB2 from *Cuphea hookeriana* preferred C10-ACP to C8-ACP with ACP from *E. coli*, while converting preference to C8-ACP with ACP from *Cuphea lanceolate*. Circular dichroism (CD) results indicated that the C8-EcACP encountered a 34.4% α-helix increment compared to C10-EcACP, which resulted in an approximate binding affinity decrease in ChFatB2 compared to C10-EcACP. Similarly, the C10-ClACP2 suffered a 45% decrease in helix content compared to C8–ClACP2, and the conformational changes resulted in an 18% binding affinity decline with ChFatB2 compared with C10-ClACP2. In brief, the study demonstrates that the ACP portion of acyl-ACP contributes to the selectivity of acyl-ACP thioesterase, and the conformational changes of EcACP and ClACP2 switch the chain length preference of ChFatB2 between C8 and C10. The result provides fundamentals for the directed synthesis of medium-chain fatty acids based on regulating the conformational changes of ACPs.

## 1. Introduction

Microbial fatty acids are promising biofuel precursors, especially medium-chain (C8–C12) fatty acids and their derivatives, which have been in significant demand for chemical products and aviation fuels [1,2]. However, the fatty acids produced by microorganisms and plants are usually palmitic acid (C16) and stearic acid (C18), or their derivatives [3,4]. These fatty acids are de novo synthesized by the Type II fatty acid synthase (FAS) system with the acyl initially loaded onto the acyl carrier protein (ACP), elongated by the fatty acid synthase, and finally offloaded by acyl-ACP thioesterase (TE), which catalyzes the termination of acyl-ACP elongation [5]. Thus, tuning the FAS-related enzymes is expected to be an effective strategy for tailoring the chain length of the fatty acid products [6,7,8].

The specificity of acyl-ACP TEs has been identified as playing a crucial role responsible for acyl chain length, which is mainly determined by the acyl-binding cavity of the enzyme [9,10]. Reshaping the hydrophobic cavity with bulkier or more hydrophobic residues resulted in a smaller acyl-binding cavity that better accommodated shorter acyl chains [11], while reshaping the hydrophobic cavity that binds the acyl substrate with smaller side chains such as T137G of UcFatB at the bottom of the pocket proved to be an effective way to produce long-chain fatty acids [12]. In addition to the reconstruction of substrate cavities, recent studies have proved that the protein-to-protein interactions between acyl-ACP TE and ACP are functional in determining the chain length as well as the fatty-acid yield. Engineering the interaction interfaces of acyl-ACP TEs with ACP by replacement of two positively charged residues on the AbTE surface proved to be highly effective with more than a three-fold improvement in the production of secreted medium-chain fatty acids [13]. Similarly, engineering the interaction interface’s positively charged surface patches with CvFatB2 may facilitate binding of the ACP moiety, which has resulted in improving the shorter chains [11]. Recently, overexpressed acyl-CoA thioesterase TesA and all fatty acid synthase in *E. coli* proved that the enzyme concentration was a parameter in fatty acid synthase systems to control the fatty acid product profiles [14]. Moreover, by exchanging sequence fragments encoding the regions between acyl-ACP thioesterases with different substrate preferences, the chimeric thioesterases demonstrated substantial changes to alter chain length specificity [15].

In addition to the specificity of acyl-ACP thioesterases, the acyl carrier proteins, which usually share the canonical four-helix fold, have been demonstrated to present dynamic structural conformations depending on the acyl chain length. They act as the central protein that delivers the acyl substrates to partner enzymes. ACP consists of Helix I being antiparallel to Helices II and IV, plus a short 3_10_ or α-helical segment (Helix III) linking Helices II and IV. A chain-flipping mechanism between ACP and its partner enzymes was proposed using pantetheine probes attached to ACP, indicating the structural changes of acyl-ACPs during the reaction [16,17].

Several details of the variable structure of ACP contributing to the recognition of TE have been identified, such as the way microalgal ACPs may not cooperate with plant TEs [18], while expression of the heterologous *Dunaliella tertiolecta* fatty acyl-ACP TE, with more homology to *Chlamydomonas reinhardtii,* leads to increased lipid production [19]. Additionally, the hydrophobic binding cavity of ACP was identified to accommodate different chain lengths [20]. Based on the structural characteristics of ACP, the modification of the EcACP cavity by I75W and I75Y, to increase the steric hindrance of the acyl chain, enhanced the production of medium-chain fatty acids [21]. The results indicate particularly that engineering of flexible ACP could be combined with TEs affecting length specificity in the synthesis of fatty acids. However, the relationship between the specificity of TE and the conformational changes of ACP has been seldom studied.

Limited to the preparation of acyl-ACPs, most interaction studies on ACP and acyl-ACP TE are based on *E. coli* cellular levels [22,23], which may be affected by the partner enzymes of ACP and the carbon flow distribution of acyl-ACPs [24]. To further clarify the chain length selectivity between acyl-ACP TE and acyl-ACP, here, two ACPs are selected, one from *Escherichia coli* (EcACP) and the other from *Cuphea lanceolate* (ClACP2). Based on enzymatic studies in vitro, it is demonstrated that the selectivity of medium-chain specific ChFatB2 from *Cuphea hookeriana* prefers C10-EcACP to C8-EcACP, but this preference could be converted from C8-ClACP2 to C10-ClACP2. Differing from the perspective that acyl-ACP TE mainly determines the preferences of the acyl chain, it is proposed that the different conformations of ACP directly contribute to the chain length selectivity of TE, which is further proved by secondary structure analysis and protein-protein interaction studies.

## 2. Results

### 2.1. Structure and Sequence Comparison of ACPs from Different Species

Previously, engineering ChFatB2 from *Cuphea hookeriana* into *E. coli* presented an accumulation of octanoic acid and capric acid, indicating molecular recognition between ChFatB2 and EcACP. It was suggested that ACPs from higher plants would be more adaptive relative to EcACP based on an homologous relationship to plant ChFatB2. The plastid-derived ACPs from higher plants usually consist of 130–140 amino acids with an N-terminal chloroplast transit peptide, and the remaining parts are structured similar to *E. coli* ACP. Here, ClACP2 from an homologous *Cuphea lanceolata* was elected in this study because the gene sequence of ACP from *Cuphea hookeriana* has not been reported. The sequence similarity between ClACP2 and EcACP was 38.46%, while ClACP2 was more homologous to ACP from *Spinacia oleracea* with 65.85% sequential similarity. The latter has been deeply studied and could be used as a structural model.

**Figure 1:**
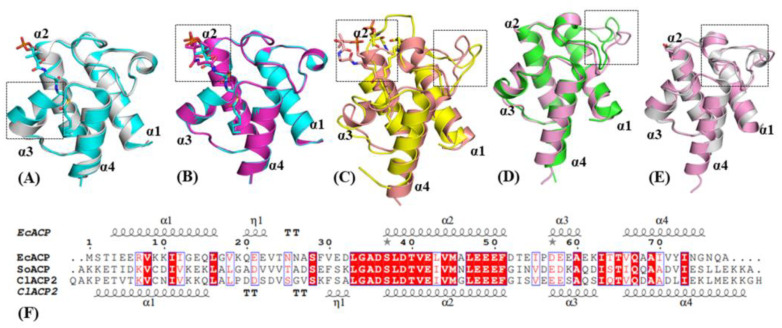
Comparisons of the structures and sequences of ACPs. (**A**) Structure overlay of EcACP by apo form (PDB entry 1T8K, colored gray) and C7-acylation form (PDB entry 2FAD, colored cyan). (**B**) Structure overlay of EcACP by C7-acylation form and C10-acylation form (PDB entry 2FAE, colored purple). (**C**) Structure overlay of SoACP by C10-acylation form (PDB entry 2FVF colored salmon) and C18:0-acylation form (PDB entry 2FVA, colored yellow). (**D**) Overlay of SoACP (PDB entry 2FVE, colored green) and ClACP2 (colored pink). (**E**) Overlay of EcACP by apo form and ClACP2. (**F**) Sequence comparison of EcACP, SoACP, and ClACP2. The red boxes show the 100% conserved residues; residues having similar properties are shown in blue boxes.

Dozens of ACPs from *E. coli* and plants have been resolved by crystal and NMR structures. Representative conformational changes of ACP have been identified that may play functional roles in the accommodation of acyl and molecular recognition with partner enzymes. The apo form of EcACP folds as the typical four α-helixes, while the C7 acylation on phosphopantetheine triggers a compact folding with the α3 helix turning closer to the acyl portion (Figure 1A). Compared with C7-EcACP, the C10-EcACP showed slight changes in the acyl-binding region, while most changes were found in the phosphopantetheine portion to adapt the acyl chain (Figure 1B). As for longer acyl chains, the ACPs would conduct substantial conformational changes by the loop between the α1–α2 region other than the acyl binding pocket, as confirmed in the C18:0-SoACP and C10:0-SoACP (Figure 1C). Structure modeling of ClACP2 indicated a similar fold with SoACP, except the loop region between α1–α2 (Figure 1D), where the sequence was not conserved (Figure 1F). In comparing ClACP2 with EcACP (Figure 1E), a η-helix was formed between the α1–α2 helix with residues 19–22, and the ClACP2 was more likely to form a helix with residues 30–33. The results indicated that the ACPs presented conformational changes induced by different acyl chains and the ACP portion, which may affect the recognition of the partner enzymes.

### 2.2. ChFatB2 Prefers ClACP2 to EcACP as the Acyl Carrier

**Figure 2:**
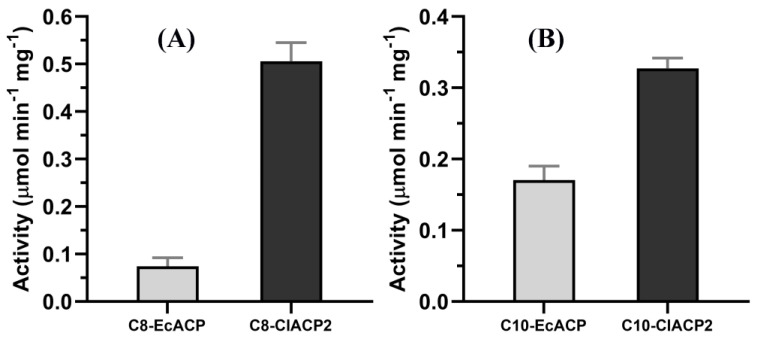
Relative activity of ChFatB2 with acyl-ACPs. (**A**) Relative activity of ChFatB2 with C8-EcACP and C8-ClACP2 as the substrate. (**B**) Relative activity of ChFatB2 with C10-EcACP and C10-ClACP2 as the substrate.

Regarding the molecular recognition of ChFatB2 by acyl-ACPs, particularly on the ACP portion, EcACP and ClACP2 excluding the chloroplast transit peptide were expressed in the *E. coli* BAP1 strain. C8 and C10 acyl-ACPs using EcACP and ClACP2 as the acyl carrier were synthesized by acyl-ACP synthetase in vitro. Then the relative activity of ChFatB2 on acyl-EcACP and acyl-ClACP2 was compared, as shown in Figure 2. The relative activity of ChFatB2 was 0.07 ± 0.01 µmol min^−1^ mg^−1^ towards C8-EcACP and 0.51 ± 0.04 µmol min^−1^ mg^−1^ towards C8-ClACP2. In addition, the activity of ChFatB2 against C10-EcACP and C10-ClACP2 was 0.17 ± 0.02 µmol min^−1^ mg^−1^ and 0.33 ± 0.01 µmol min^−1^ mg^−1^, respectively. The results suggested that ChFatB2 showed 7.29 higher fold activity using C8-ClACP2 as substrate rather than C8-EcACP, and 1.94 fold activity increase using C10-ClACP2 rather than C10-EcACP. As expected, the enzymatic activity of different ACPs revealed that ChFatB2 preferred ClACP2 from higher plants with a closer relationship to EcACP.

### 2.3. Kinetic Parameters of ChFatB2 with Acyl-ACPs

As far as the preference for ACPs, the results suggested that ChFatB2 preferred C10-EcACP and C8-ClACP2. Then the kinetic constants including *K*_m_, *k*_cat_, and *k*_cat_/*K*_m_ of ChFatB2 for C8-EcACP and C10-EcACP were calculated and shown in Table 1. The ChFatB2 presented *k*_cat_ to C8-EcACP with 1.38 ± 0.09 min^−1^ while presenting to C10-EcACP with 2.16 ± 0.23 min^−1^. Moreover, ChFatB2 presented *K*_m_ to C8-EcACP with 349.5±66.5 µM and to C10-EcACP with 185.7 ± 40.5 µM. Then the C10-EcACP preference was defined as C10(*k*_cat_/*K*_m_)/C8(*k*_cat_/*K*_m_), and the specific value was 2.94, indicating the significant preference of ChFatB2 for C10-EcACP.

Furthermore, the kinetic constants of ChFatB2 for C8-ClACP2 and C10-ClACP2 were determined. ChFatB2 presented *k*_cat_ to C8-ClACP2 with 6.36 ± 0.51 min^−1^ and to C10-EcACP with 5.64 ± 0.32 min^−1^. In addition, ChFatB2 presented *K*_m_ to C8-ClACP2 with 216.7 ± 49.9 µM while to C10-ClACP2 with 263.1 ± 30.9 µM. Contrary to that of acyl-EcACP, the C8-ClACP2 preference was defined as C8(*k*_cat_/*K*_m_)/C10(*k*_cat_/*K*_m_), and the specific value was 1.37, indicating the apparent preference of ChFatB2 for C8-ClACP2.

### 2.4. CD Spectra Results Reveal the Conformational Changes of the Acyl-ACPs

**Figure 3:**
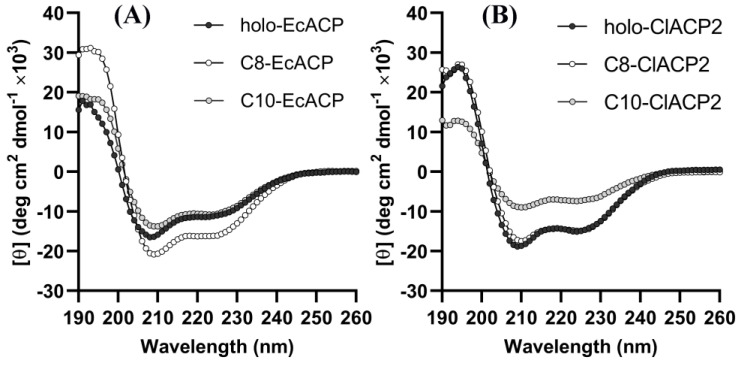
Circular dichroism spectra of the ACPs (**A**) holo-, C8-, and C10-EcACP from 190 to 260 nm wavelength. (**B**) holo-, C8-, and C10-ClACP2 from 190 to 260 nm wavelength. The mean residue ellipticity (θ) in degrees cm^2^ dmol^−1^.

The enzymatic results clearly demonstrated that the acyl preference of ChFatB2 switched from C8 to C10 when using ClACP2 as the carrier protein instead of EcACP and suggested that modification of the ACP portion would contribute to the acyl chain preference of the thioesterase. It was assumed that the conformational changes of the entire acyl-ACP, particularly the changes of the ACP portion, were the principal reason. Due to the unique and conserved helix structure of ACP, circular dichroism provides a reliable scheme for studying the structural changes. The double minima at 208 nm and 222 nm were determined for all holo- and acyl-ACPs from 190 to 260 nm, as shown in Figure 3, confirming the characteristics of the α-helix structure in the CD spectra.

Furthermore, the α-helix content and changes of the ACPs were calculated using the Dichroweb program, as shown in Table 2. The holo-ACP was calculated to contain 54.9 ± 3.1% α-helices, which was consistent with the crystal structure data. Similarly, the C10-EcACP presented 51.2 ± 1.3% α-helices, while the C8-EcACP showed a helix content of 73.8 ± 1.1% compared to the holo-ACP, indicating an apparent structural change with α-helices increased by 34.4%. With regard to ClACP2, the α-helix content of holo-ClACP2 was calculated to be 68.9 ± 0.1%, which was approximately identical to that of C8-ClACP2 at 67.3 ± 1.1%. Whereas the α-helix content of C10-ClACP2 significantly reduced to 37.5 ± 0.3%, the results indicated that large conformational changes occurred in C10-ClACP2 instead of C8-ClACP2.

### 2.5. The Thermal Denaturation Measurements of the ACPs

**Figure 4:**
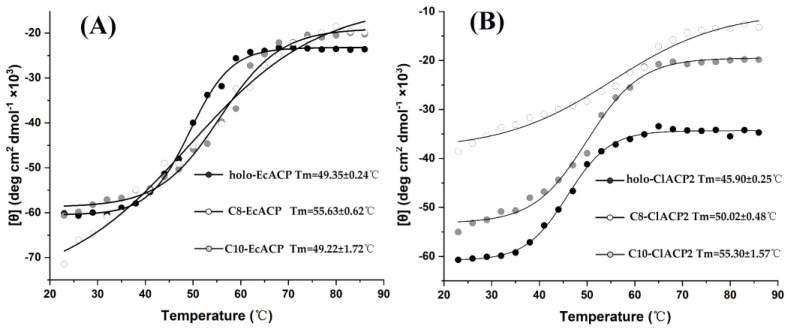
Melting temperature curves of ACP. (**A**) The thermal-induced melting temperature curves of holo-, C8- and C10-EcACP. (**B**) The thermal-induced melting temperature curves of holo-, C8- and C10-ClACP2.

To further confirm the structural differences of holo-, C8-, and C10-ACP, the melting temperatures of the ACPs were determined based on thermotropic assay as shown in Figure 4. The holo-EcACP showed a Tm of 49.35 ± 0.24 °C, which was similar to that of C10-EcACP at 49.22 ± 1.72 °C. However, the C8-EcACP that had undergone α-helix content changes presented a Tm of 55.63 ± 0.62 °C. With regard to ClACP2, the holo-ClACP2 showed a Tm of 45.90 ± 0.25 °C, which was closer to that of C8-ClACP2 with 50.02 ± 0.48 °C, while the C10-ClACP2 increased the Tm to 55.30 ± 1.57 °C. The results were in agreement with the α-helix contents of the ACPs determined by CD spectra and indicated that the structure of C8-EcACP and C10-EcACP, with respect to C8-ClACP2 and C10-ClACP2, undergoes significant structural changes, which may take effect upon the interaction with partner enzymes and the chain length selectivity of the enzymes.

### 2.6. Binding Affinity of ChFatB2 with the ACPs Determined by Liquid Crystal Assay

The CD spectra showed that C10-EcACP revealed subtle structural changes relative to holo-EcACP, while C8-EcACP revealed significant secondary structural changes. These results were similar to the kinetic constants, which showed that ChFatB2 preferred C10-EcACP instead of C8-EcACP. The same circumstances were found in ClACP2, that ChFatB2 preferred C8-ClACP2, consistent with holo-ClACP2. Therefore, it was proposed that the recognition of ChFatB2 with acyl-ACPs was associated with the structural characteristics of holo-ACPs. The binding affinity of ChFatB2 with the acyl-ACPs has been determined by Michaelis constants, while the interaction with holo-ACP was unavailable. Isothermal titration calorimetry method was introduced but failed due to the weak reaction heat. Liquid crystals with a special physical and optical property can be used to report chemical and biomolecular binding events (such as the binding of the target protein and the ligand) through their orientational behaviors and the corresponding optical textures [25,26,27]. Then the semi-quantitative method was applied to investigate their interactions.

**Figure 5:**
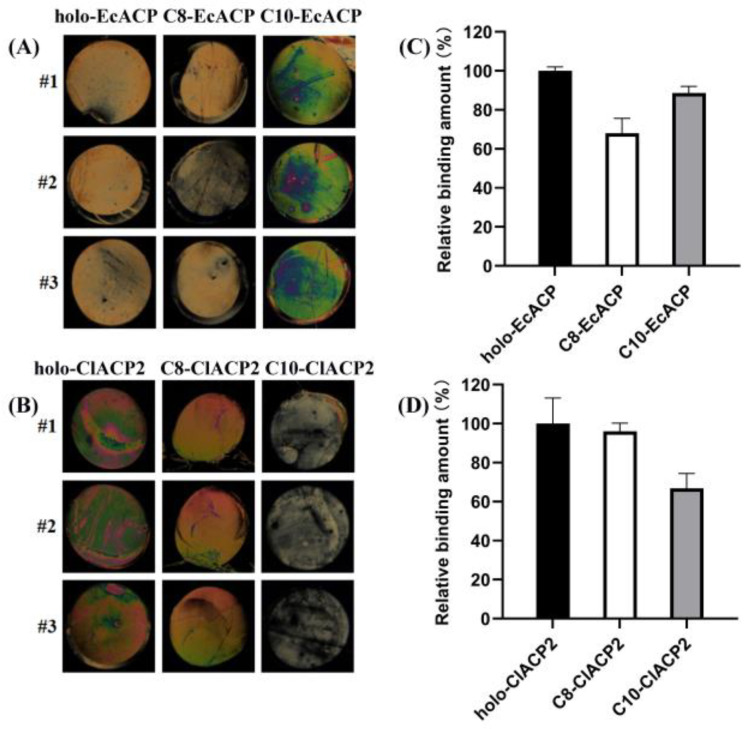
Binding affinity of ChFatB2 with the ACPs determined by liquid crystals assay. (**A**,**B**) show the optical micrographs of a thin layer of liquid crystal under polarized microscope. The liquid crystals were sandwiched between a DMOAP-coated glass slide and a DMOAP-coated glass slide with circular domain of immobilized ChFatB2 and EC-holo-ACP, C8-, and C10-EcACP (**A**), and immobilized ChFatB2 and holo-ClACP2, C8-ClACP2, and C10-ClACP2 (**B**). (**C**,**D**) are the grayscale values of the optical images of (**A**,**B**) obtained by Image J software. The analysis was repeated three times (#1, #2, and #3).

The immobilized ChFatB2 was incubated with different acyl- and holo-ACPs. After rinsing, the optical images of liquid crystal cells in Figure 5A,B showed that the different circular regions display different optical brightness under polarized light microscopy. This indicated that the orientations of liquid crystals in different regions were different, which may be caused by the amount of absorbed ACPs. Because the immobilized ChFatB2 was the same for each circular region, the difference of the absorbed ACPs may be attributed to their binding capability with ChFatB2. The relative binding capacity of ChFatB2 to ACP was evaluated by the grayscale of the optical image of liquid crystal cells shown in Figure 5C,D. The average of the grayscale in the holo-EcACP-deposited region was 155.7 ± 3.2 and defined as 100% binding with ChFatB2. The grayscale values in C8-EcACP- and C10-EcACP-deposited regions were 106.1 ± 11.8 and 138.1 ± 5.3, respectively, and corresponded to 68.1% and 88.6%, compared with the holo form. Compared to the binding of C10-EcACP and holo-EcACP with ChFatB2, the C8-EcACP with conformational changes presented lower binding, which is consistent with the study above. With regard to ClACP2, the grayscales of the optical images in holo-, C8-, and C10-deposited regions were 144.7 ± 19.1, 139.1 ± 6.0, and 96.7 ± 11.0 and calculated to 100%, 96.1%, and 66.8% binding, respectively. Similarly, the C10-ClACP2 that carries conformational changes presented a decline in binding compared to C8-ClACP2 and holo-ClACP2.

## 3. Discussion

The structures of several ACPs in apo-form and acyl-forms have been resolved, with the acyl chains stored in a hydrophobic pocket formed by four α-helices. Even though the ACPs consist of similar folds, some flexible regions were identified, especially residues 19–38 in the α1–α2 loop region. The sequence and structure comparison showed that a specific η-helix including residues 32–35 was observed for the structures of ClACP2 and SoACP in the α1–α2 loop region, but this was not apparent in the structures of holo-EcACP and C10-EcACP or the α1–α2 loop region located at the entrance of the acyl binding cavity. Molecular dynamics simulations of the acyl forms of EcACP revealed that the hydrophobic binding pocket of ACP is best suited to accommodate a C8 group and was capable of adjusting the size to accommodate a C10 acyl group [28]. Additionally, the conformation of the α1–α2 loop region was also variable for different chain lengths of acyl-ACP. The docking results of EcACP and UcFatB from *Umbellularia californica* indicated that the α1–α2 loop region of EcACP was involved in the recognition with UcFatB [12]. Another structural model of EcACP and EcTesA showed that residue D32 in the α1–α2 loop region of EcACP interacted with R77 of EcTesA [29]. Therefore, the η-helix region between the α1–α2 region of ClACP2 may play positive roles in recognition with thioesterases from plants. Thus, it was supposed that the recognition of ChFatB2 with ClACP2 and with EcACP would be significantly different.

Due to the valuable prospects of medium-chain fatty acids, engineering the chain length preference of acyl-ACP thioesterase has attracted much attention. To date, most efforts to control fatty acid synthesis in engineered microbes have focused on modifying termination of acyl-ACP thioesterases, and mutagenesis efforts have succeeded in tuning free fatty acid product profiles and identifying the key features driving substrate specificity and activity of the acyl-ACP thioesterase [30,31]. However, in some circumstances, given the unnatural recognition between the exogenous medium-chain-specific thioesterases and the endogenous ACP, production of fatty acids is limited. The regulation of endogenous ACP may provide a new strategy for directed synthesis of medium-chain fatty acids. Using EcACP and ClACP2 as the acyl carrier, enzymatic data showed that the activity of ChFatB2 against the substrate C8-ClACP2 was 6.8-fold higher than that of C8-EcACP, and 1.6-fold higher than that of C10-EcACP against C10-ClACP2. And ChFatB2 showed higher activity against ClACP2 for both C8 and C10 substrates, which indicated a stronger recognition between ChFatB2 and ClACP2 than EcACP. Furthermore, the enzymatic kinetic constants of ChFatB2 for EcACP and ClACP2 were determined, respectively. The binding affinity of ChFatB2 for C10-EcACP was higher than that of C8-EcACP, and the *k*_cat_/*K*_m_ of the substrate C10-EcACP was 2.92-fold higher than that of C8-EcACP, which indicated that ChFatB2 preferred the C10 substrate for EcACP. Interestingly, the *k*_cat_/*K*_m_ of ChFatB2 for C8-ClACP2 was 1.26-fold higher than that of C10-ClACP2. It was indicated that ChFatB2 preferred the C8 substrate of ClACP2, while previously it preferred the C10 substrate of EcACP. These findings can also be analyzed from the data of other investigators that for C16:0 and C18:0 chain length ACP substrates, HaFatB preferred C16:0-EcACP, while previously it preferred C18:0-HaACP2 [32]. Therefore, it can be deduced that the chain length preference of the thioesterase is not only determined based on its characteristics, but the ACP portion also makes direct contributions to the selectivity of the enzyme.

These results are different from the conventional understanding that thioesterases primarily determine the chain length of fatty acid products. We speculate that this is caused by structural differences in the ACPs that affect the recognition of the thioesterase. To further investigate the mechanism of ACP influence on the chain length preference of ChFatB2, the secondary structure of these ACPs was analyzed by CD spectroscopy. The data suggests that the introduction of acyl chains affects the secondary structure of EcACP and ClACP2. This is similar to the previous findings that for C4:0-EcACP, C6-EcACP, C7-EcACP, and C10-EcACP, varying size and complexity of chains, including the conformation of the phosphopantetheine linker, participated in recognizing the interacting enzymes [20]. The largest changes in the structure were located in the α2–α3 loop, α3, and α3–α4 loop regions. After the introduction of the acyl chains, the acyl chains were inserted into the hydrophobic core of the protein, which triggered the movement of several hydrophobic residues arranged in the cavity, thus affecting the conformation of the ACP. Moreover, the conversion of holo-SoACP to C10-SoACP and C18-SoACP was accompanied by different conformational changes. The hydrophobic cavities of C10-SoACP and C18-SoACP were 157 Å^3^ and 228 Å^3^, respectively, and the most obvious change was the disappearance of the α–helix in the α1–α2 ring region of C18-SoACP [33].

On the other hand, the CD results showed a change of +34.4% in the secondary structure for C8-EcACP relative to holo-EcACP, while C10-EcACP showed minor changes. Relative to holo-ClACP2, C10-ClACP2 showed +45.5% changes in the secondary structure content, while C8-ClACP2 showed slight changes. The comprehensive results of the CD results and kinetic experiments indicated that the secondary structures of C10-EcACP and C8-ClACP2 were preferred by ChFatB2. We speculate that this change in structure relative to the allosteric ACP may affect the chain length preference of ChFatB2 by influencing the secondary structure of the ACP and thus the recognition with ChFatB2. Based on this, it was proposed that the recognition affinity of ChFatB2 with holo-EcACP should be similar to C10-EcACP but stronger than C8-EcACP, while the opposite would be found for C8-ClACP2 and C10-ClACP2.

The binding affinity of ChFatB2 and acyl-ACP can be reflected through the Michaelis constants; however, interaction of ChFatB2 with the product holo-ACPs was seldom reported. Previous research studied the binding affinity between several mutants of acyltransferase VinK and VinL-ACP using the isothermal titration calorimetry (ITC) assay [34]. However, we found that the ITC assay was not suitable, due to the weak binding heat change between ChFatB2 and ACPs. Studies showed that the concentration of the ligands bound to a particular receptor could be successfully determined semi-quantitatively using liquid crystal analysis. The amount of glucose specifically recognized with glucose oxidase was determined by liquid crystal assay [35], and the concentration of glycoprotein gp120 specifically recognized with it was detected using B40t77 aptamer as a molecular probe [36]. Similarly, the molecular interactions between these ACPs and ChFatB2 were compared by liquid crystal assays. The data showed that ChFatB2 was capable of recognizing C10-EcACP, with a proximate binding affinity to holo-EcACP, while taking into consideration the C8-ACP that suffered 34.4% structural changes; the binding affinity declined to 47% and caused the lower activity of ChFatB2 to C8-EcACP. The same results occurred in the ClACP2 as acyl carrier: that the 45% structural changes of C10-ClACP2 led to an 18% binding affinity compared to holo-ClACP2. The results were in agreement with the supposition that the conformation changes of acyl-ACP affects intermolecular interactions with ChFatB2 and contributes to the chain length preference of ChFatB2.

## 4. Materials and Methods

### 4.1. Bacterial Strains and Materials

ChFatB2 excluding the transit peptide (residues 129–395) was constructed on the pCold II vector and transformed into *E. coli* BL21 (DE3) for protein expression, as described previously [24]. For the expression of holo-ACP, pET28a_EcACP was transferred into the host strain BAP1, which contained a chromosomal insertion of the Bacillus subtilis Sfp gene, as described [37]. Moreover, pET28a_ClACP2 (Uniport No. P52412) with residues 54–137 was expressed in consistency with EcACP. Chemical reagents including ampicillin, kanamycin, Bradford, and DTT are purchased from Sangon Biotech in Shanghai, China.

### 4.2. Structure and Sequence Comparison of the ACPs

The structure of ClACP2 was modeled using the AlphaFold Protein Structure Database with the high confidence they were calculated from residues 54–137 as overexpressed in *E. coli*. Sequence comparison of the ACPs was generated using the Clustw2 program and analyzed using Espript 3.0 [38]. The red boxes show the 100% conserved residues, while residues having similar properties are shown in blue boxes.

### 4.3. Protein Expression and Purification

The *E. coli* strains carrying the recombinant ChFatB2 and ACP vectors were cultured in Luria-Bertani (LB) broth containing 50 μg/mL ampicillin and Kanamycin, respectively, and induced by 0.5 mM Isopropyl-β-D-thiogalactopyranoside (Takara Biomedical Technology (Beijing) Co., Beijing, China) when the cells had grown to OD_600_ of 0.5 at 37 °C. The cells were further grown at 16 °C for 12 h before being harvested by centrifugation at 6500× *g* for 20 min. The cell pellet was then suspended in a lysis buffer (50 mM Tris-HCl pH 8.0, 300 mM NaCl, 5% glycerol (*v*/*v*), and 1 mM β-mercaptoethanol) and homogenized by ultrasonication on ice. The lysate was centrifuged for 30 min at 12,000× *g* at 4 °C. The supernatant containing soluble target protein was then applied onto Ni-NTA resin (QIAgen, Hilden, Germany). The eluted proteins were further purified on a Superdex 200 column (Cytiva, Marlborough, MA, USA) equilibrated with Tris-HCl buffer (50 mM Tris-HCl pH 8.0, 500 mM NaCl, 5% glycerol (*v*/*v*), 1 mM DTT). The fractions containing ChFatB2 were concentrated using an Amicon concentrator 10 kDa (Merck Millipore, Darmstadt, Germany), while ACPs were concentrated using 3 kDa, and the protein concentrations were determined using Bradford assay with BSA as standard.

### 4.4. Synthesis of Acyl-ACPs

Acyl-ACPs were prepared using an acyl-ACP synthetase (AaaS) from *Vibrio harveyi*. Each 100 μL reaction assay contained 25 mM Tris-HCl, 2 mM DTT, 5 mM MgCl2, 10 mM ATP, 50 μM holo-acyl-carrier-protein, 100 mM fatty acid (8:0, 10:0), and 0.1 μg purified AaaS protein, and holo-ACP was completely converted to acyl-ACP at 30 °C for 90 min. Then the acyl-ACPs were purified with Superdex 200 column and stocked at a concentration of 11 mg^−1^ mL^−1^.

### 4.5. HPLC Analysis of Acyl-ACPs

For holo-ACP and (C8:0, C10:0)-ACP analysis and identification, a 10 μL diluted sample (0.2 mg^−1^ mL^−1^) was loaded onto a C8-ODS column on the HPLC(Agilent 1260, Waldbronn, Germany). Holo-ACP and (C8:0, C10:0)-ACP were eluted with 0.1% trifluoroacetic acid (Thermo Fisher Scientific, Waltham, MA, USA) at a linear gradient of 65–35 and 35–65% acetonitrile (Thermo Fisher Scientific, Waltham, MA, USA) for 20 min at a flow rate of 1 mL min^−1^. Acyl-ACP and holo-ACP were detected at an absorption of 210 nm.

### 4.6. Enzymatic Activity for the Thioesterase ChFatB2

Thioesterase kinetic activity was measured by diagnosis of the sulfhydryl content released as a product of the reaction, as previously reported [39]. The reaction mixture was composed of 100 mM phosphate buffer pH 8.0, 1 mM 5,5′-Dithiobis- (2-nitrobenzoic acid) (DTNB). The final volume was 100 μL. The absorbance value at 412 nm was measured after 5 min. The activity made known as moles of acyl-ACP hydrolysed per minute per milligram was calculated using ε_412_ = 13,600 M^−1^ cm^−1^. Each assay was measured with three separate replicates, and the mean values were estimated. The data from ChFatB2 assays were fitted to the Michaelis–Menten equation by nonlinear least-square regression analysis using GraphPad Prism 8 software.

### 4.7. Circular Dichroism Measurements of the ACPs

CD measurements were performed by a Bio-Logic MOS-500 spectrometer using a cell with a 1-mm path length. The protein concentrations were 0.12–0.38 mg mL^−1^ and were dissolved in 10 mM phosphate buffer pH 8.0. The CD spectra of the ACPs were collected from 190 to 260 nm at 0.1 nm intervals at 25 °C. The mean values from three scans were averaged and smoothed, processed with CDToolX- Windows 10 Version software, and plotted as the mean residue ellipticity (θ) in deg cm^2^ dmole^−1^. The Dichroweb software COSSTR program was used to analyze the content of the α-helix, β-Turns, and unordered secondary structure [40]. For thermal denaturation measurements, the ellipticity was measured at 222 nm with 3 °C increments from 20 °C to 86 °C, at a rate of 90 °C/h. Thermal melting (Tm) points were calculated with a Boltzmann sigmoidal fit using OriginLab OriginPro 2019.

### 4.8. The Liquid Crystal Assay of ChFatB2 and the ACPs

The DMOAP-coated glass slides were prepared as described [22]. First, a few drops of ChFatB2 (5 μg mL^−1^, 1.5 µL), which led to the homotropic alignments of liquid crystals, were applied on the DMOAP-coated glass slides. After drying, the acyl-ACP solutions with different conformations (10 μg mL^−1^, 1.5 µL) were deposited on the circular regions with immobilized ChFatB2. After 30 min of incubation, the slides were washed with deionized water and dried under nitrogen flow. The sample slide was then assembled into a liquid crystal cell and observed under a polarized microscope (Nikon eclipse LV100 POL, Tokyo, Japan). The greyscale values of the optical images were calculated by using Image J software 1.46r.

## 5. Conclusions

In conclusion, a novel approach of manipulating the acyl carrier protein portion of acyl-ACP to control the chain length propensity of the thioesterase is reported. Instead of engineering the selectivity of acyl-ACP thioesterases to govern the fatty acid profile, it was proved that C8-EcACP and C10-EcACP present significant conformational changes and are directly involved in the interaction with the thioesterase. Similar characteristics were found in C8-ClACP2 and C10-ClACP2, while the different ACPs switched the chain length preference of ChFatB2 between C8-ClACP2 and C10-EcACP. To meet the growing demand of medium-chain fatty acids in biofuels and fine chemicals, this result would provide fundamentals for directed synthesis of medium-chain fatty acids based on regulating the conformations of ACPs.

## Figures and Tables

**Table 1:** The enzymatic constants of ChFatB2 with acyl-ACPs.

	C8-EcACP	C10-EcACP	C10-EcACP Preference ^(a)^	C8-ClACP2	C10-ClACP2	C8-ClACP2 Preference ^(b)^
*K_m_* (µM)	349.5 ± 66.5	185.7 ± 40.5	-	216.7 ± 49.9	263.1 ± 30.9	-
*k_cat_* (min^−1^)	1.38 ± 0.09	2.16 ± 0.23	-	6.36 ± 0.51	5.64 ± 0.32	-
*k_cat_*/*K_m_* (µM^−1^ min^−1^)	3.95 × 10^−3^	11.6 × 10^−3^	2.94	29.4 × 10^−3^	21.4 × 10^−3^	1.37

^(a)^ The C10-EcACP preference was defined as C10(*k_cat_*/*K_m_*)/C8(*k_cat_*/*K_m_*), and ^(b)^ The C8-ClACP2 preference was defined as C8(*k_cat_*/*K_m_*)//C10(*k_cat_*/*K_m_*).

**Table 2:** The secondary structure contents of acyl-ACPs calculated by Dichroweb program.

Protein	α-helix (%)	Turns (%)	Unordered (%)	Relative Change (%) ^(a)^
holo-EcACP	54.9 ± 3.1	12.1 ± 1.7	31.5 ± 2.5	0
C8-EcACP	73.8 ± 1.1	8.9 ± 0.9	17.2 ± 0.5	+34.4
C10-EcACP	51.2 ± 1.3	14.3 ± 1.6	30.6 ± 2.4	−6.7
holo-ClACP2	68.9 ± 0.1	10.1 ± 0.8	20.4 ± 1.2	0
C8-ClACP2	67.3 ± 1.1	9.1 ± 1.0	23.1 ± 2.2	−2.3
C10-ClACP2	37.5 ± 0.3	15.6 ± 1.7	33.0 ± 0.9	−45.5

^(a)^ The relative changes (%) were calculated using the α-helix content of holo-EcACP and holo-ClACP2 as 100%, respectively.

## Data Availability

All relevant data are within the paper.
